# COVID-19 Vaccination Coverage and Associated Factors in Patients with Multiple Sclerosis

**DOI:** 10.3390/vaccines12020126

**Published:** 2024-01-26

**Authors:** Ignacio Hernández-García, Joana Rodríguez-Montolio, Monserrath Almeida-Zurita, Dionisio Cheli-Gracia, Belén del Moral Sahuquillo, Carlos Aibar-Remón, Moisés Garcés-Redondo

**Affiliations:** 1 Department of Preventive Medicine and Public Health, Lozano Blesa University Clinical Hospital of Zaragoza, Calle San Juan Bosco 15, 50009 Zaragoza, Spain; 2 Health Services Research Group of Aragon (GRISSA), Aragon Institute for Health Research (IISA), Calle San Juan Bosco 15, 50009 Zaragoza, Spain; caibar@unizar.es; 3 Department of Neurology, Lozano Blesa University Clinical Hospital of Zaragoza, Calle San Juan Bosco 15, 50009 Zaragoza, Spain; jrodriguezm@salud.aragon.es (J.R.-M.); pmalmeida@salud.aragon.es (M.A.-Z.); dcheli@salud.aragon.es (D.C.-G.); bdelmoral@salud.aragon.es (B.d.M.S.); mgarcesr@salud.aragon.es (M.G.-R.); 4 Department of Microbiology, Pediatrics, Radiology and Public Health, University of Zaragoza, Calle de Pedro Cerbuna 12, 50009 Zaragoza, Spain

**Keywords:** COVID-19 vaccines, multiple sclerosis, vaccination coverage, COVID-19 full primo-vaccination, COVID-19 booster dose, associated factors, vaccination strategies

## Abstract

Our objective was to know the COVID-19 vaccination coverage in multiple sclerosis (MS) patients and its factors associated. A retrospective cohort study was carried out. Patients seen at the MS unit of the University Clinical Hospital of Zaragoza between 2017 and 2021 were included. Variables were obtained by reviewing the specialized and primary care records. Associations between receiving COVID-19 full primo-vaccination, as well as one booster dose since autumn 2022, and the other variables were analyzed using bivariate analysis and multiple logistic regression models. Of the 359 included patients, 90.3% received the COVID-19 full primo-vaccination. Having been born in Spain (_a_OR = 3.40) and having received the 2020–2021 influenza vaccine (_a_OR = 6.77) were associated with receiving the COVID-19 full primo-vaccination. Vaccination with a COVID-19 booster dose was detected in 141 patients (39.3%). Sex (man) (_a_OR = 2.36), age (60 years or over) (_a_OR = 6.82), type of MS (Primary Progressive/Secondary Progressive) (_a_OR = 3.94), and having received the 2022–2023 influenza vaccine (_a_OR = 27.54) were associated with receiving such a booster dose. The COVID-19 booster dose was administered at the same time as the 2022–2023 influenza vaccine in 57.8% (67/116) of the patients vaccinated with both vaccines. The COVID-19 full primo-vaccination coverage is higher than in other countries. However, the decrease in vaccination coverage with the booster dose makes it necessary to develop strategies to improve it that are not limited to administering the flu vaccine together with the COVID-19 booster dose. Such strategies should be in focus, especially for women under 60 years of age.

## 1. Introduction

Multiple sclerosis (MS) is an autoimmune demyelinating inflammatory disease that affects an estimated 2.8 million people worldwide (35.9 per 100,000 population) [1]. People with this disease are at increased risk for respiratory infections [2,3]; they also often require disease-modifying therapies (DMT), which by immunosuppressing or altering their immune system further increase their risk for upper respiratory tract infections [4,5]. All this is particularly relevant, as infections in these patients are associated with the onset or worsening of baseline MS symptoms in the form of relapses or pseudo-relapses [6]. In particular, upper respiratory tract infections may double their risk of relapses [7], and it has even been claimed that relapses associated with an infection may be more neurologically damaging than those not related to an infection [8]. Thus, measures to prevent respiratory infections are especially important for these individuals, with vaccination being the most important of these measures.

Since the onset of the COVID-19 pandemic caused by the new Severe Acute Respiratory Syndrome Coronavirus 2 (SARS-CoV-2), persons with MS have been considered “extra” vulnerable due to the immune-mediated nature of their disease, disability status, and immunomodulating treatments [9]. Indeed, to date, SARS-CoV-2 infection-related mortality rates of up to 4% have been reported in these individuals [10,11]; furthermore, higher rates of infection and hospitalization due to COVID-19 have been reported in MS patients than in the general population [12].

In this context, SARS-CoV-2 vaccines, which represent the most important preventive measure for the control of this pandemic, are safe and effective in MS patients [13,14,15] even though certain drugs (e.g., anti-CD20 therapies) can decrease the immune response to vaccines [14]; for this reason, many organizations recommend the vaccination thereof [16,17]. In particular, in the Spanish COVID-19 vaccination strategy [18,19] (Table 1), MS people are among the population groups in which, due to the Omicron variant’s emergence, both the Spanish Ministry of Health and the General Directorate of Public Health of Region of Aragon have recommended the administration of one booster dose of mRNA COVID-19 vaccines since autumn 2022 [18,19,20]; for this purpose, free of charge vaccines are inoculated generally in primary care centers [19,20].

A fundamental element of any vaccination strategy is its evaluation. In this regard, the Spanish Ministry of Health periodically publishes the results of COVID-19 vaccination coverage achieved in the general population by age group and Autonomous Community [22]. However, to our knowledge, COVID-19 vaccination coverage obtained specifically in patients with MS has not yet been evaluated in Spain, in contrast to what has recently occurred in other countries, such as Austria [23], Switzerland [24], Australia [25], or Iran [26]. Specifically, COVID-19 vaccination rates of 87.0% (in Austria) [23], 91.4% (in Switzerland) [24], 82.9% (in Australia) [25], and 88.3% (in Iran) [26] have been reported in MS patients. Among the few studies about the factors that influence MS patients’ decision to get vaccinated against COVID-19, misinformation on infectious diseases and vaccines was reported to be associated with lower vaccination rates [23].

This research was carried out with the objective of knowing the COVID-19 vaccination coverage in patients with MS in our environment and the factors associated with it.

## 2. Materials and Methods

### 2.1. Study Design and Setting

A cross-sectional study in the Region of Aragon (Spain) was carried out; about 1,200,000 people live in this Spanish Region. The Lozano Blesa University Clinical Hospital of Zaragoza (LBUCHZ) houses one of the two MS units in the Region of Aragon. In it, patients are monitored at least every six months.

### 2.2. Patients and Inclusion Criteria

MS patients who were seen at such a unit from 1 January 2017 to 31 December 2021 were eligible for inclusion (since it was the estimated time needed to obtain a sample of 323 persons, which corresponded to the sample size for a confidence level of 95%, a precision of 5%, and an expected COVID-19 vaccination coverage of at least 70% [27]). The exclusion criteria were death or relocation of their place of residence outside Aragon before 26 September 2022 (the date on which the COVID-19 booster dose began to be administered in Spain [19,20]).

### 2.3. Variables and Data Collection

In August 2023, physicians of the Department of Neurology of the LBUCHZ compiled the following information by reviewing patients’ electronic medical records: year of birth, sex, country of birth, area of residence (rural or urban), allergies, age at MS diagnosis, MS type (according to the American Academy of Neurology) [28], number of MS outbreaks during 2020 and 2021, Expanded Disability Severity Scale (EDSS) in 2020 and 2021 [29], flu vaccination status in 2018–2019, 2019–2020, 2020–2021, 2021–2022, and 2022–2023, name of COVID-19 vaccine(s) received, date(s) of administration, having been diagnosed with COVID-19 by laboratory confirmation test (i.e., detection of viral RNA or antigenic detection by oropharyngeal or nasal swab) during the 12 months prior to the start of the vaccination campaign with the COVID-19 booster dose, and belonging to any other target group subject to vaccination with the COVID-19 booster dose according to the recommendations of the Spanish Ministry of Health/General Directorate of Public Health of Region of Aragon [19,20] (persons 60 years or older, persons less than 60 years who are at high risk of complications by COVID-19 (chronic cardiovascular disease, chronic respiratory disease, chronic neurological diseases, diabetes mellitus, morbid obesity, chronic kidney disease or nephrotic syndrome, hemoglobinopathies and anemias, hemophilia, other coagulation disorders and chronic bleeding disorders, recipients of blood products and multiple transfusions, asplenia or severe asplenic dysfunction, chronic liver disease, severe neuromuscular diseases, immunosuppression, cancer, cochlear implantation or awaiting cochlear implantation, cerebrospinal fluid fistula, celiac disease, Crohn’s disease, ulcerative colitis, systemic erythematosus lupus, rheumatoid or juvenile arthritis, Down Syndrome, dementia)’, persons institutionalized in nursing homes or chronic care centers and pregnant women in any gestational trimester).

### 2.4. Statistical Analysis

A descriptive analysis of all variables was performed, using measures of central tendency (mean or median) and dispersion (standard deviation or range) for quantitative variables and absolute and relative frequencies (percentages) for qualitative variables. The following definitions were used to describe COVID-19 vaccination coverage [18,20,30]: (a) full primo-vaccination: 2 doses of mRNA vaccines (Comirnaty (by Pfizer/BioNTech (New York, NY, USA)) or Spikevax (by Moderna (London, UK))), 2 doses of Vaxzevria adenovirus vector vaccine (by Astrazeneca/Oxford (Oxford, UK)), 1 dose of Vaxzevria adenovirus vector vaccine (by Astrazeneca/Oxford (Oxford, UK)) plus 1 dose of mRNA vaccine (by Comirnaty), or 1 dose of Janssen adenovirus vector vaccine (by Johnson & Johnson (New Brunswick, NJ, USA)); (b) vaccination with 1 booster dose against COVID-19: full primo-vaccination plus 1 booster dose of mRNA vaccines (by Pfizer/BioNTech (New York, NY, USA) or Moderna (London, UK)) administered since 26 September 2022 (date on which this booster dose began to be administered in Spain [19,20]).

In addition, two bivariate analyses were performed in which having received COVID-19 full primo-vaccination and full primo-vaccination plus 1 booster dose against COVID-19 were considered as dependent variables. The chi-square test or, if appropriate, Fisher’s exact test was used to quantify the associations. Subsequently, two multiple logistic regression analyses were carried out with the variables for which a significant association was detected in the bivariate analyses. To quantify the associations, the adjusted odds ratio (_a_OR) was obtained with its 95% confidence intervals (95% CIs).

A statistical significance level of *p* < 0.05 was established. All of this was carried out using the analysis program IBM SPSS Statistics 26.0.

### 2.5. Ethical Considerations

This study was approved by the Research Ethics Committee of the Region of Aragon (protocol code C.I. EPA23/043). In addition, with respect to the publication of the results of this research, the authors adhered to the guidelines of the Code of Good Research Practices of the University of Zaragoza (Spain) [31].

## 3. Results

After excluding 10 patients who died or relocated their place of residence outside Aragon between 2017 and 26 September 2022, the number of patients studied was 359. A total of 63.0% (226/359) were women, with a median age of 31 years (range: 10–62 years) at diagnosis. Of the patients, 93.6% were born in Spain. According to MS type, 82.2% presented the relapsing–remitting type. No patient presented an allergy to the components of the COVID-19 or influenza vaccines. At the beginning of the primo-vaccination, the mean age (standard deviation) of the patients was 44.7 (12.3) years, and at the time of receiving the booster dose, it was 46.4 (12.3) years. A total of 91.4% (328/359) belonged to at least one other target group for the COVID-19 booster dose (the most frequent was immunosuppressive treatment in 270 patients) (Table 2).

Among the patients studied, the first COVID-19 vaccine was administered on 6 January 2021. The number of patients who received at least one dose of COVID-19 vaccine was 333 (92.8%). Of the patients, 90.3% (324/359) received the COVID-19 full primo-vaccination; in particular, 88.6% received two doses of mRNA vaccines as primo-vaccination (Table 2). By sex, full primo-vaccination coverage was 90.3% in women and 90.2% in men. In patients born in Spain, COVID-19 full primo-vaccination coverage was 91.9%, and in those born outside of Spain, it was 65.2%. Such coverage remained between 97.1% and 97.8% in persons with a history of having received the influenza vaccine in any of the campaigns carried out between 2018 and 2020.

In the bivariate analyses, the variables that were significantly associated with having received COVID-19 full primo-vaccination were: (a) number of years from MS diagnosis (10 years or over); (b) country of birth (Spain), (c) having chronic cardiovascular disease, and (d) having a history of flu vaccination in the 2018–2019, 2019–2020, or 2020–2021 seasons (Table 3).

In the logistic regression analysis, the variables that maintained their significant association were as follows: having been born in Spain (_a_OR (95%CI) = 3.40 (1.26–9.21)) and having received the 2020–2021 influenza vaccine (_a_OR (95%CI) = 6.77 (2.28–20.11)) (Table 4).

Among the patients studied, the COVID-19 booster dose was administered for the first time on 26 September 2022 and for the last time on 13 April 2023. Vaccination with such a booster dose was detected in 141 patients (39.3%); according to sex, vaccination coverage was 46.6% in men and 34.9% in women. Such coverage was 41.1% in patients born in Spain and 13.0% in those born outside of Spain. According to influenza vaccination history, coverage with the COVID-19 booster dose ranged from 54.4% to 74.8% in persons who had received a flu vaccine in the 2020–2021 and 2022–2023 seasons, respectively.

In particular, the COVID-19 booster dose was administered at the same time as the 2022–2023 influenza vaccine in 57.8% (67/116) of the patients who were vaccinated with both vaccines.

In the bivariate analyses, the variables associated with vaccination with the COVID-19 booster dose were: (a) sex (man), (b) age (60 years or over), (c) number of years from MS diagnosis (10 or over), (d) country of birth (Spain), (e) MS type (Primary Progressive/Secondary Progressive), (f) EDSS score (6.5 or higher), (g) history of influenza vaccination in the 2018–2019, 2019–2020, 2020–2021, 2021–2022, or 2022–2023 seasons, (h) not taking immunosuppressive treatment, and (i) having a chronic cardiovascular disease (Table 5).

In the multivariate analyses, the variables that maintained a statistically significant association were as follows: (a) sex (man) (_a_OR (95%CI) = 2.36 (1.13–4.93)), (b) age (60 years or over) (_a_OR (95%CI) = 6.82 (1.94–23.95)), (c) type of MS (Primary Progressive/Secondary Progressive) (_a_OR (95%CI) = 3.94 (1.29–11.95)), and (d) having received the 2022–2023 influenza vaccine (_a_OR (95%CI) = 27.54 (12.56–60.37)) (Table 6).

## 4. Discussion

This research is the first in Spain to study COVID-19 vaccination coverage in MS patients. Both the percentage of patients who received at least one dose of the COVID-19 vaccine (92.8%) and the full primo-vaccination rate (90.3%) are higher than that obtained by other authors in other countries [23,24,25,26,32,33], such as Croatia, where COVID-19 full primo-vaccination rates of 64.4% have been reported in MS patients [32].

However, it is not possible to make valid comparisons with the coverages obtained in other research carried out in the rest of the countries, such as Austria (87.0%) [23], Iran (88.3%) [26], Australia (82.9%) [25], Switzerland (91.4%) [24], or Canada/United States of America (USA) (84.1%) [33], because these studies only evaluated whether MS patients had received at least one dose of the COVID-19 vaccine, without specifying that they had received the full primo-vaccination schedule.

COVID-19 full primo-vaccination coverage is also higher than that obtained in the general population in Spain (86.0%) [34], especially with respect to certain groups of people in particular, such as fruit workers (78.7%) [35] or persons infected with the human immunodeficiency virus (HIV) (66.6%) [36]. On the other hand, this full primo-vaccination coverage was lower than that obtained in Spain among health students (97.8%) [37] and primary care professionals (95.2%) [38]. Perhaps, occupational COVID-19 exposure, which is a variable that has been described as being independently associated with the acceptance of the COVID-19 vaccine by healthcare workers may contribute to explaining these differences [39].

With respect to other groups of patients in Spain, such as inflammatory bowel disease patients, COVID-19 vaccination coverage with at least one dose was similar to that obtained in our patients (94.4% [40]). In turn, this vaccination coverage was higher than that obtained in patients with chronic diseases in countries such as Ethiopia (29.6% [41]) or the USA (75.6% [42]). Among the reasons that could explain these higher vaccination coverages than in other countries is the fact that Spaniards are very supportive and gregarious people, in the sense that they understand that there are things that must be done not for themselves but for others. An example, before vaccines, is that of transplants, where Spain has been a leader for decades [43]. In addition, the population has a very good relationship with the healthcare system because it is a public and universal healthcare system that can be trusted on a regular basis and there are no anti-vaccine movements [43].

In our research, the finding that persons born in Spain have significantly higher COVID-19 full primo-vaccination rates than those born in other countries constitutes health inequality [44]. Given its unfairness [44], it is very necessary to carry out specific strategies to improve COVID-19 full primo-vaccination coverage in persons born outside of Spain. In this regard, several studies have reported that increasing the population’s knowledge about disease severity and the benefits of vaccines could improve their willingness to be vaccinated [45,46]. Therefore, public health strategies should focus on providing adequate information to the public in general, and to persons born outside of Spain in particular, both on the disease and on the evidence of the efficacy and safety of COVID-19 vaccines.

With the onset of the COVID-19 pandemic, an increase in the 2020–2021 influenza vaccination coverage (of almost 13 percentage points with respect to the 2019–2020 season) was observed in our patients. This increase has also been described by other authors in studies carried out in our country on healthcare workers [38] and has been attributed to the need to avoid a co-infection of influenza and SARS-CoV-2, in a context of lack of knowledge of the possible consequences in case of a possible co-infection [38].

Also, similar associations between having received the 2020–2021 influenza vaccine and being COVID-19 fully primo-vaccinated have been described in other studies [37]. This association could be explained by both greater attention to recommendations and greater concern for one’s own health, which translates into greater compliance [37].

On the other hand, despite evidence that a COVID-19 booster dose restores a degree of protection against infection and hospitalization caused by the SARS-CoV-2 similar to the initial two doses [47], the vaccination rate with the COVID-19 booster dose administered since September 2022 decreased to 39.3%. Given that studies on COVID-19 vaccination in MS patients were conducted before September 2022 [23,24,25,26,32,33], our research is the first to report vaccination coverage with such a booster dose among these patients.

Furthermore, to our knowledge, no data have yet been published in Spain on vaccination coverage with this COVID-19 booster dose, which is why we can only make comparisons with the results of studies published at the international level in other groups of people. For example, in Italy, COVID-19 booster dose vaccination rates of 18.8% have been reported among medical residents of a University Hospital between October to December 2022 [48]; this value has been attributed to the misinformation campaigns that have been carried out during the COVID-19 pandemic and to the ever more present anti-vaccination movements, both leading to the rise of vaccine hesitancy. In the USA, a COVID-19 booster dose vaccination rate of 27.3% has been reported in pregnant women between October 2022 and April 2023 [49]. Thus, one might suppose that the higher coverage obtained in our research could perhaps be due to the fact that we had collected data in August 2023, but, as specified above, the last booster dose recorded in our patients was administered on 13 April 2023; likewise, if those administered since 1 January 2023 are discarded (36 doses), the resulting booster dose vaccination rate in our patients up to 31 December 2022 (29.3% (105/359)) is still higher than that obtained in Italy.

In any case, there is much room for improvement in vaccination coverage with the COVID-19 booster dose, and therefore strategies for its improvement should be implemented that are not limited to co-administering this vaccine with the influenza vaccine, given that in our study only 57.8% (67/116) of the patients who were vaccinated with both vaccines received them simultaneously. Among other possible strategies for improving vaccination coverage are the following: (a) medical reminders (i.e., messages sent to patients to remind them of the importance of being vaccinated with the COVID-19 booster dose; such messages could be sent by autodialed phone calls, post-cards, or text messages) [50,51], (b) promoting vaccination by all physicians, including patients’ neurologists [52] and primary care physicians (since it has been documented with other vaccines, such as influenza, that receiving medical advice to vaccinate is an effective method of increasing flu vaccination rates [53]), (c) offering vaccination as a default option during patient visits and integrating vaccination into medical practice procedures [50], (d) combating misinformation [50], (e) disseminating information on the effectiveness of the vaccine (COVID-19 full primo-vaccination, plus the booster dose against COVID-19, reduces the risk of hospitalization for COVID-19 by up to 94% and the risk of severe forms of illness of COVID-19 infections by up to 87% [54]), and (f) disseminating specific information on vaccination recommendations in MS patients [50,51] (given that, so far, in the posters used in health centers in Aragon, or on the SaludInforma web portal of the Aragon Department of Health [55], the information provided to citizens on the COVID-19 booster dose is somewhat generic (without specifying the need for patients with MS, or with chronic neurological diseases in general, to receive the COVID-19 booster dose). The effectiveness of implementing these measures could be the subject of future research.

On the other hand, other measures implemented in other countries to try to improve vaccination coverage with the COVID-19 booster dose, such as conditional cash lotteries [56,57], are not considered to be applicable in our country, given the existing culture in Spain that vaccination is an act of self-protection and solidarity at the same time [58]. In fact, in Spain, although no vaccines are compulsory, there is a general awareness that they are beneficial for the people who receive them and for society as a whole [58].

Like other authors, we detected a decrease in influenza vaccination coverage after the 2020–2021 season [48,59], a fact that would confirm the trend toward cessation of the positive effect that the COVID-19 pandemic had on the influenza vaccination coverage obtained in 2020–2021 [60]. Among the variables associated with receiving the COVID-19 booster dose, having received the 2022–2023 influenza vaccine was detected; this association could again be explained both by greater attention to recommendations and greater concern for one’s own health, which translates into greater compliance. On the other hand, being a woman and being younger than 60 years of age were associated with not having received the booster dose, which allows us to detect a typology of patients in whom interventions to increase their vaccination rate should be prioritized.

Prior to this, specific research should be carried out to find out the reasons for not having been vaccinated, taking into account that, with the results of our study, some of them could be ruled out, such as being allergic to the vaccine (since no patient was allergic to the vaccine) or having had a positive case of COVID-19 in the last few months, reflecting the belief that natural immunity protects against COVID-19 infection [37,61] (because this variable was not associated with receiving the booster dose).

There are some limitations of the present study. The sample size (359), similar to or larger than that of other studies (281 [25], 367 [26]), may have contributed to not very accurate results (with wide confidence intervals). Like other authors, our research was carried out in a single region of one country [23,26,35,36,48,62]; never less, we provide a systematic approach to the evaluation of COVID-19 vaccination rates in MS patients that could be carried out in other MS units. Furthermore, despite having analyzed the possible association of various factors with vaccination against COVID-19, it is necessary to complement this study with some qualitative research to analyze the possible association between vaccination against COVID-19 and the knowledge, beliefs, and attitudes of patients about this disease and its vaccine. On the other hand, our study presents strong points, such as that there were no missing data about vaccinations, because such information is always recorded in the electronic medical record. Moreover, our research is the first to provide information on the results of the COVID-19 vaccination strategy developed in Spain in persons with MS. It shows an alarming decrease in vaccination coverage with the booster dose, which demonstrates the need to implement measures for its improvement that complement the offer of this vaccine with the influenza vaccine. One possible improvement measure would be to offer brief medical advice to vaccinate the subgroups of people with MS that we detected as less vaccinated.

## 5. Conclusions

The COVID-19 full primo-vaccination rate in MS patients has been very high; however, vaccination coverage with the COVID-19 booster doses suffered an important decrease. Improvement interventions should focus on primo-vaccination in patients born outside of Spain, as well as on vaccination with the booster dose in women under 60 years of age. For all of this, it will be necessary to integrate different strategies that are not limited to administering the flu vaccine together with the COVID-19 booster dose.

## Figures and Tables

**Table 1:** Evolution of the COVID-19 full primo-vaccination strategy in Spain [18,21].

		Vaccines Used
**December 2020–January 2021** **(first available doses)**	Primo-vaccination priority groups: *Residents and staff in nursing homes and care centers for the elderly and those with high dependency rates* *Front-line health and social-health personnel* *Other health and social-health personnel* *Non-institutionalized major dependents*	2 doses of Comirnaty mRNA vaccine (by Pfizer/BioNTech (New York, NY, USA)) or 2 doses of Spikevax mRNA vaccine (by Moderna (London, UK))
**February 2021** **(more available doses)**	Other primo-vaccination priority groups: *Older than 80* *People between 70 and 79 and people with very high-risk conditions* *People between 60 and 65* *People between 66 and 69* *Other health and social-health personnel* *Workers with an essential social function* *People between 50 and 59*	2 doses of Comirnaty mRNA vaccine (by Pfizer/BioNTech (New York, NY, USA)) or 2 doses of Spikevax mRNA vaccine (by Moderna (London, UK)) or 2 doses of Vaxzevria adenovirus vector vaccine (by Astrazeneca/Oxford (Oxford, UK))
**March 2021**		2 doses of Comirnaty mRNA vaccine (by Pfizer/BioNTech (New York, NY, USA)) or 2 doses of Spikevax mRNA vaccine (by Moderna (London, UK)) or 2 doses of Vaxzevria adenovirus vector vaccine (by Astrazeneca/Oxford (Oxford, UK)) or 1 dose of Janssen adenovirus vector vaccine (by Johnson & Johnson (New Brunswick, NJ, USA))
**May 2021**		2 doses of Comirnaty mRNA vaccine (by Pfizer/BioNTech (New York, NY, USA)) or 2 doses of Spikevax mRNA vaccine (by Moderna (London, UK)) or 2 doses of Vaxzevria adenovirus vector vaccine (by Astrazeneca/Oxford (Oxford, UK)) or 1 dose of Janssen adenovirus vector vaccine (by Johnson & Johnson (New Brunswick, NJ, USA)) or 1 dose of Vaxzevria vaccine plus 1 dose of Comirnaty vaccine
**June 2021** **(vaccine widely available)**	Other primo-vaccination priority groups: *People between 40 and 49* *People between 30 and 39* *Persons between 20 and 29* *Persons between 12 and 19*	2 doses of Comirnaty mRNA vaccine (by Pfizer/BioNTech (New York, NY, USA)) or 2 doses of Spikevax mRNA vaccine (by Moderna (London, UK)) or 2 doses of Vaxzevria adenovirus vector vaccine (by Astrazeneca/Oxford (Oxford, UK)) or 1 dose of Janssen adenovirus vector vaccine (by Johnson & Johnson (New Brunswick, NJ, USA)) or 1 dose of Vaxzevria vaccine plus 1 dose of Comirnaty vaccine
**December 2021**	Other primo-vaccination groups: *People between 5 and 11*	Primo-vaccination in persons aged 5 to 11 years with 2 doses of pediatric Comirnaty mRNA vaccine (by Pfizer/BioNTech (New York, NY, USA))
**February 2022**		Nuvaxovid protein subunit vaccine (by Novavax (Gaithersburg, MD, USA)) for persons 18 years of age and older who have not been able to be vaccinated due to allergy to mRNA vaccines components

**Table 2:** Results of the descriptive analysis.

	*N* = 359
**Sex, *n (%)***	
Woman	226 (63.0)
Man	133 (37.0)
**Median age at multiple sclerosis diagnosis (range), *years***	31 (10–62)
**Area of residence, *n (%)***	
Urban	188 (52.4)
Rural	171 (47.6)
**Country of birth, *n (%)***	
Spain	336 (93.6)
Romania	6 (1.7)
Morocco	4 (1.1)
Algeria	3 (0.8)
Others	10 (2.8)
**Multiple sclerosis type, *n (%)***	
Relapsing–Remitting	295 (82.2)
Secondary Progressive	57 (15.9)
Primary Progressive	7 (1.9)
**Influenza vaccination coverage, *n (%)***	
2018–2019	127 (35.4)
2019–2020	138 (38.4)
2020–2021	184 (51.3)
2021–2022	173 (48.2)
2022–2023	155 (43.2)
**COVID-19 primo-vaccination schedule, *n (%)***	
2 doses of mRNA vaccines	287 (88.6)
2 doses of Vaxzevria adenovirus vector vaccine	21 (6.5)
1 dose of Janssen adenovirus vector vaccine	12 (3.7)
1 doses of doses of Vaxzevria adenovirus vector vaccine plus 1 doses of mRNA vaccine	4 (1.2)
**COVID-19 diagnosis in the previous 12 months to the start of the vaccination campaign with the COVID-19 booster dose, *n (%)***	
Yes	120 (33.4)
No	239 (66.6)
**Belonging to any other target group subject to vaccination with the booster dose against COVID-19, *n (%)***	
Yes	328 (91.4)
No	31 (8.6)

Results expressed as absolute (n) and relative (%) frequencies.

**Table 3:** Results of the bivariate analysis for COVID-19 full primo-vaccination.

	COVID-19 Full Primo-Vaccination		*p*
	Yes (*n* = 324)	No (*n* = 35)	
**Sex, *n (%)***			
Man	120 (37.0)	13 (37.1)	0.990
Woman	204 (63.0)	22 (62.9)	
**Age, *n (%)***			
60 years or over	37 (11.4)	2 (5.7)	0.401
Under 60 years	287 (88.6)	33 (94.3)	
**Number of years from MS diagnosis, *n (%)***			
10 years or over	161 (53.7)	11 (33.3)	0.027
Under 10 years	139 (46.3)	22 (66.7)	
**Country of birth, *n (%)***			
Spain	309 (95.4)	27 (77.1)	0.001
Others	15 (4.6)	8 (22.9)	
**Area of residence, *n (%)***			
Urban	168 (51.9)	20 (57.1)	0.552
Rural	156 (48.1)	15 (42.9)	
**Multiple sclerosis type, *n (%)***			0.298
Relapsing–Remitting	264 (81.5)	31 (88.6)	
Primary Progressive/Secondary Progressive	60 (18.5)	4 (11.4)	
**Multiple sclerosis outbreaks in 2020, *n (%)***			
1 or more	40 (14.6)	3 (11.5)	1.000
None	234 (85.4)	23 (88.5)	
**Expanded Disability Severity Scale score in 2020, *n (%)***			
6.5 or higher	30 (58.5)	1 (77.3)	0.689
3.5–6	44 (7.5)	3 (5.3)	1.000
1.5–3	73 (34.0)	8 (17.4)	0.616
0–1	106 (34.0)	9 (34.0)	
**Flu vaccination in 2018–2019, *n (%)***			<0.001
Yes	124 (38.3)	3 (8.6)	
No	200 (61.7)	32 (91.4)	
**Flu vaccination in 2019–2020, *n (%)***			
Yes	134 (41.4)	4 (11.4)	0.001
No	190 (58.6)	31 (88.6)	
**Flu vaccination in 2020–2021, *n (%)***			<0.001
Yes	180 (55.6)	4 (11.4)	
No	144 (44.4)	31 (88.6)	
**Immunosuppressive treatment, *n (%)***			
Yes	248 (76.5)	22 (62.9)	0.075
No	76 (23.5)	13 (37.1)	
**Cancer, *n (%)***			
Yes	12 (3.7)	1 (2.9)	1.000
No	312 (96.3)	34 (97.1)	
**Chronic respiratory disease, *n (%)***			1.000
Yes	14 (4.3)	1 (2.9)	
No	310 (95.7)	34 (97.1)	
**Chronic kidney disease, *n (%)***			1.000
Yes	6 (1.9)	0 (0)	
No	318 (98.1)	35 (100)	
**Diabetes mellitus, *n (%)***			0.402
Yes	15 (4,6)	3 (8.6)	
No	309 (95.4)	32 (91.4)	
**Chronic cardiovascular disease, *n (%)***			0.007
Yes	57 (17.6)	0 (0)	
No	267 (82.4)	35 (100)	
**Celiac disease, *n (%)***			1.000
Yes	5 (1.5)	0 (0)	
No	319 (98.5)	35 (100)	
**Inflammatory bowel disease, *n (%)***			1.000
Yes	3 (0.9)	0 (0)	
No	321 (99.1)	35 (100)	
**People in disability centers or nursing homes, *n (%)***			1.000
Yes	3 (0.9)	0 (0)	
No	321 (99.1)	35 (100)	

**Table 4:** Variables included in the multiple logistic regression model for COVID-19 full primo-vaccination.

	COVID-19 Full Primo-Vaccination		_a_OR (95%CI)	*p*
	Yes (*n* = 324)	No (*n* = 35)		
**Country of birth, *n (%)***				
Spain	309 (95.4)	27 (77.1)	3.40 (1.26–9.21)	0.016
Others	15 (4.6)	8 (22.9)	1	
**Flu vaccination in 2020–2021, *n (%)***				
Yes	180 (55.6)	4 (11.4)	6.77 (2.28–20.11)	0.001
No	144 (44.4)	31 (88.6)	1	

_a_OR (95%CI): adjusted odds ratio (95% confidence interval).

**Table 5:** Results of the bivariate analysis for vaccination with 1 booster dose against COVID-19 since September 2022.

	Vaccination with 1 Booster Dose against COVID-19		*p*
	Yes (*n* = 141)	No (*n* = 218)	
**Sex, *n (%)***			
Man	62 (44.0)	71 (32.6)	0.029
Woman	79 (56.0)	147 (67.4)	
**Age, *n (%)***			<0.001
60 years of age or over	43 (30.5)	18 (8.3)	
Under 60 years of age	98 (69.5)	200 (91.7)	
**Number of years from MS diagnosis, *n (%)***			
10 years or over	88 (68.8)	105 (51.2)	0.002
Under 10 years	40 (31.2)	100 (48.8)	
**Country of birth, *n (%)***			
Spain	138 (97.9)	198 (90.8)	0.008
Others	3 (2.1)	20 (9.2)	
**Area of residence, *n (%)***			
Urban	68 (48.2)	120 (55.0)	0.206
Rural	73 (51.8)	98 (45.0)	
**Multiple sclerosis type, *n (%)***			<0.001
Relapsing–Remitting	100 (70.9)	195 (89.4)	
Primary Progressive/Secondary Progressive	41 (29.1)	23 (10.6)	
**Multiple sclerosis outbreaks in 2021, *n (%)***			
1 or more	8 (6.2)	28 (13.9)	0.064
None	122 (93.8)	174 (86.1)	
**Expanded Disability Severity Scale score in 2021, *n (%)***			
6.5 or higher	24 (19.8)	17 (8.9)	0.005
3.5–6	21 (17.4)	29 (15.2)	0.294
1.5–3	33 (27.3)	60 (31.4)	0.770
0–1	43 (35.5)	85 (44.5)	
**Flu vaccination in 2018–2019, *n (%)***			
Yes	77 (54.6)	50 (22.9)	<0.001
No	64 (45.4)	168 (77.1)	
**Flu vaccination in 2019–2020, *n (%)***			
Yes	76 (53.9)	62 (28.4)	<0.001
No	65 (46.1)	156 (71.6)	
**Flu vaccination in 2020–2021, *n (%)***			
Yes	100 (70.9)	84 (38.5)	<0.001
No	41 (29.1)	134 (61.5)	
**Flu vaccination in 2021–2022, *n (%)***			
Yes	102 (72.3)	71 (32.6)	<0.001
No	39 (27.7)	147 (67.4)	
**Flu vaccination in 2022–2023, *n (%)***			
Yes	116 (82.3)	39 (17.9)	<0.001
No	25 (17.7)	179 (82.1)	
**Immunosuppressive treatment, *n (%)***			
Yes	95 (67.4)	175 (80.3)	0.006
No	46 (32.6)	43 (19.7)	
**Cancer, *n (%)***			
Yes	6 (4.3)	7 (3.2)	0.605
No	135 (95.7)	211 (96.8)	
**Chronic respiratory disease, *n (%)***			0.055
Yes	2 (1.4)	13 (6.0)	
No	139 (98.6)	205 (94.0)	
**Chronic kidney disease, *n (%)***			1.000
Yes	2 (1.4)	4 (1.8)	
No	139 (98.6)	214 (98.2)	
**Diabetes mellitus, *n (%)***			0.339
Yes	9 (6,4)	9 (4.1)	
No	132 (93.6)	209 (95.9)	
**Chronic cardiovascular disease, *n (%)***			<0.001
Yes	37 (26.2)	20 (9.2)	
No	104 (73.8)	198 (90.8)	
**Celiac disease, *n (%)***			1.000
Yes	2 (1.4)	3 (1.4)	
No	139 (98.6)	215 (98.6)	
**Inflammatory bowel disease, *n (%)***			1.000
Yes	1 (0.7)	2 (0.9)	
No	140 (99.3)	216 (99.1)	
**People in disability centers or nursing homes, *n (%)***			0.060
Yes	3 (2.1)	0 (0)	
No	138 (97.9)	218 (100)	
**COVID-19 diagnosis in the previous 12 months, *n (%)***			0.102
Yes	40 (28.4)	80 (36.7)	
No	101 (71.6)	138 (63.3)	
**COVID-19 diagnosis in the previous 6 months, *n (%)***			0.112
Yes	19 (28.4)	18 (36.7)	
No	122 (71.6)	200 (63.3)	

**Table 6:** Variables included in the multiple logistic regression model for 1 booster dose against COVID-19 since September 2022.

	1 Booster Dose against COVID-19		_a_OR (95%CI)	*p*
	Yes (*n* = 141)	No (*n* = 218)		
**Sex, *n (%)***				
Man	62 (44.0)	71 (32.6)	2.36 (1.13–4.93)	0.023
Woman	79 (56.0)	147 (67.4)	1	
**Age, *n (%)***				
60 years of age or over	43 (30.5)	18 (8.3)	6.82 (1.94–23.95)	0.003
Under 60 years of age	98 (69.5)	200 (91.7)	1	
**Multiple sclerosis type, *n (%)***				
Primary Progressive/Secondary Progressive	41 (29.1)	23 (10.6)	3.94 (1.29–11.95)	0.016
Relapsing–Remitting	100 (70.9)	195 (89.4)	1	
**Flu vaccination in 2022–2023, *n (%)***				
Yes	116 (82.3)	39 (17.9)	27.54 (12.56–60.37)	<0.001
No	25 (17.7)	179 (82.1)	1	

_a_OR (95%CI): adjusted odds ratio (95% confidence interval).

## Data Availability

Data are contained within the article.
